# Assessing predictors of self-management intentions in people with type 2 diabetes

**DOI:** 10.1186/s12913-022-07731-x

**Published:** 2022-03-21

**Authors:** Yogarabindranath Swarna Nantha, Tan Yan Shan, Shamsul Haque, Anuar Zaini Md Zain

**Affiliations:** Clinical School Johor Bahru, Monash University Malaysia, No. 8, Masjid Sultan Abu Bakar, 80100 Johor Bahru, Johor Malaysia

**Keywords:** Self-management behaviour, Type 2 diabetes, Theory of planned behaviour, Primary care

## Abstract

**Background:**

Type 2 diabetes mellitus (T2D) is slowly turning into an international health emergency, evidenced by accelerated growth in prevalence rates worldwide. Experts have now called for greater integration of self-management interventions in clinical practice in light of these worrisome trends, supplanting the prevailing notion of a “glucocentric” approach. In this pilot study designed to complement a novel assessment program currently in development, we describe a concise screening tool designed to stratify the intention to follow through on self-management practices in people with T2D.

**Methods:**

A cross-sectional survey was conducted at 3 regional primary care clinics. Individuals with T2D having the following characteristics were recruited into the study: (i) individuals with T2D between 18 and 65 years, (ii) fluent in English and, and iii) having been diagnosed with T2D for at least 2 years. We assessed the relevance of components in the Theory of Planned Behaviour (TPB) within the context of self-management behaviour in T2D. Participants were requested to complete a questionnaire containing questions related to intention, attitudes subjective norm and perceived behavioural control. Based on their responses, the psychometric properties of the scale were then evaluated using both reliability and validity analysis.

**Results:**

The Cronbach α value for all direct measures of TPB was excellent: intention to adhere to self-management practices (0.98), attitude towards self-management behaviour (0.87), subjective norm (0.83), and perceived behaviour control (0.66). The correlation between intentions and all 3 constructs of TPB was excellent (*p* < 0.01). Structural equation modeling helped determine attitudes and subjective norms as important predictors of intentions to follow through self-management practices.

**Conclusions:**

By first understanding the dimensions that influence intentions associated with self-management behaviour, clinicians have the opportunity to “triage” individuals with T2D who require greater involvement to bring about better self-care practices. Thus, our research attempts to bridge this gap by devising a psychometric tool suited to a regional setting which allows for an improved person-centered communication between clinicians and patients.

**Supplementary Information:**

The online version contains supplementary material available at 10.1186/s12913-022-07731-x.

## Background

For years, clinicians have adopted a conservative “glucocentric” approach to the management of diabetes [1]. Far from engendering improved health outcomes, this therapeutic policy has led to the over-treatment of diabetes, often causing more harm than benefit [2]. Experts now believe that there is a compelling reason to incorporate elements of behavioural change into pre-existing treatment strategies. In the last decade, clinical management of diabetes witnessed a gradual paradigm shift culminating in the integration of self-management interventions into routine consultations. The hallmark of this transition is characterized by 1) an active participation of patients in taking responsibility for their clinical condition, 2) an emphasis on lifestyle modifications and 3) a more holistic interpretation of target HbA1c levels [3]. The driving force behind these measures is the optimism that self-management initiatives could bring about better glycaemic control and help prevent diabetes related complications [4].

In tandem with these developments, numerous scales have been created to quantify salient dimensions associated with self-management behaviour in people with T2D. One of the earliest scale was the Summary of Diabetes Self-Care Activities- Revised (SDSCA) questionnaire, consisting of 11 items looking into self-management behaviour in participants in the week preceding the assessment [5]. Subsequently, the novel 16-item Diabetes Self Management Questionnaire (DSMQ) was created to gauge adherence to dietary and self blood glucose monitoring with greater precision [6]. Another notable self-management questionnaire is the Diabetes Management Self Efficacy Scale (DMSES) which has been widely validated in many countries [7]. Overall, these inventories were designed to evaluate self-management behaviour across a large set of individuals in the population. The interpretation of the composite scores obtained from these measurements were meant to guide meaningful public health interventions that would in return be reflected in an achievement of positive quality outcomes.

Despite these notable advances in the field of diabetes, a definitive “gold standard” for the measurement of self-management in individuals with T2D remains elusive as ever [8]. This claim is further corroborated by an analysis by Lu et al., stating that a large proportion of these inventories failed to meet robust statistical standards necessary for highly accurate questionnaires [9]. The review also declares that the quality of these questionnaires was marred by several fundamental weaknesses, which include, 1) an exclusive focus on content validity alone, 2) the lack of other forms of validity analysis (divergent and convergent validity), 3) isolated testing of the scale in a relatively homogenous population, and 4) the omission of important aspects of self-care (e.g., foot care) [9–12].

Taking stock of these methodological pitfalls in numerous studies, we have devised a comprehensive approach to evaluating self-management in people with T2D. In line with this strategy, this present article focuses on expanding the crucial principles laid out in the PRECEDE-PROCEED model [13]. Hence, the research work described here is extension of a larger study aiming to complement and reinforce a real-world approach to self-management practices in people with T2D [14]. This paper showcases the first phase of this study where we conducted a preliminary social assessment by developing a screening questionnaire that helps stratify T2D people who require urgent behavioural and treatment intervention using the TPB framework [15].

## Methods

### Participants and setting

A cross-sectional survey was conducted amongst individuals with T2D at 3 regional public primary care polyclinics between January and October 2020. According to literature, the suggested sample size for studies using the TPB framework should be more than 160 participants [16]. In this study, a total of 417 individuals with T2D were selected using a purposeful sampling strategy. A published protocol and flowchart (Supplementary File - Figure) provides a detailed description of the procedures related to this study [14].

The COVID-19 pandemic had a huge impact on our project by increasing the complexity of the data collection process. We had to employ two recruitment techniques in our study giving due consideration to all necessary safety precautions during this procedure – 1) web-based and, 2) direct participation from people with T2D attending routine clinic appointments. Identical questionnaires were used for both situations. The inclusion criteria for patient recruitment were: 1) individuals with T2D between 18 and 65 years, 2) proficiency in the English language, and 3) received a diagnosis of T2D for at least 2 years prior to the date of recruitment. Informed consent was obtained from all respondents before they participated in the study.

In the pilot phase of the study, 80 participants were tested on items related to direct measures of the TPB [16, 17]. Subsequently, an elicitation phase was conducted via qualitative interviews to ascertain elements closely linked to the TPB constructs [3–7]. These constructs govern the intention of a particular behavior: attitude, subjective norm, and perceived behavioral control (PBC) [13, 16, 17]. The questions for all three constructs (Supplementary File - Questionnaire) followed a 7-point extremely undesirable/ desirable and not important/important dimension. The mean scores for intention ranged from 1 (strongly disagree) to 7 (strongly agree). A detailed explanation about the scoring system for these constructs can be found in our published protocol paper [14].

### Statistical analysis

All acquired data were entered into the Statistical Package for Social Sciences program (SPSS version 20; IBM Corp., USA). The items with negative endpoints were recoded to reflect actual scores [16, 17]. The dataset was examined to account for missing or invalid values in order to preserve the accuracy of statistical analysis. The internal consistency of items was determined by Cronbach’s alpha values above 0.7 [18]. The reliability of the constructs was satisfactory, as determined by a statistical significance of *P* < 0.01 using the Pearson correlation analysis. Subsequently, exploratory factor analysis (EFA) was followed by structural equation modelling (SEM). These two steps were performed to assess the psychometric properties of the questionnaire and identify the overall predictive strength of each direct measurement. For all outcomes, a *P*-value < 0.05 was considered statistically significant.

## Results

### Descriptive statistics

A total of 266 (63.7%) complete responses were obtained from a total of 417 eligible participants. The primary reason for the low response can be traced back to online surveys where participants have either exited or abandoned the survey prior to completion. The participants had an average of 12.8 (SD ± 8.69) years of experience living with T2D. The mean HbA1c and BMI are 7.2% and 27.1 kg/m^2^.

The mean intention to adhere to self-management behaviour was high, with a value of 5.7 (SD ± 1.7). For the direct measures, the mean attitude [5.3, SD ± 1.5], subjective norm [6.0, SD ± 1.3], and perceived behavioural control (5.5, SD ± 1.4) recorded an average score ranging between average to good.

### Reliability of the constructs in the direct measure questionnaire

The retained items of the questionnaire were based upon an initial pilot analysis phase involving 80 participants. During that stage of the study, we analyzed and eliminated several items from each construct to improve the overall robustness of the TPB construct. As a result, the intention scale increased from 0.81 to 0.96 once two items from the construct were eliminated. Similarly, the internal consistency of the attitudes (α = 0.94 to 0.97) improved after the removal of 6 items. The robustness of the subjective norm construct rose from α = 0.67 to 0.78 when two items were removed. Also, the removal of 3 items improved the α values (from 0.70 to 0.78) of the perceived behavioural control construct.

In the formal analysis phase (involving a total of 266 responses), the Cronbach α values for intentions associated with self-management behaviour and direct measurements (Table 1) ranged from adequate to very good across the sample. The Cronbach α values were 0.98 for intentions, 0.90 for attitude (another 2 items omitted), 0.83 for subjective norms, 0.66 for perceived behavioural control.

**Table 1:** Reliability and items statistics for direct measures of self-management intentions (*n* = 266)

Constructs and items of direct measures	Mean (SD)	Cronbach Alpha	Item total correlation
**Generalized Intention**		0.98	
Item 1	5.66 (1.68)		0.96
Item 2	5.68 (1.74)		0.96
**Attitudes**		0.90	
Item 3	5.58 (1.73)		0.80
Item 4	5.30 (1.89)		0.68
Item 6	5.72 (1.75)		0.81
Item 7	5.15 (1.88)		0.73
**Subjective Norms**		0.83	
Item 9	6.02 (1.38)		0.71
Item 10	5.93 (1.44)		0.71
**Perceived Behavioural Control**		0.66	
Item 16	5.53 (1.47)		0.50
Item 17	5.45 (1.80)		0.50

### Exploratory factor analysis (EFA)

**Fig. 1:**
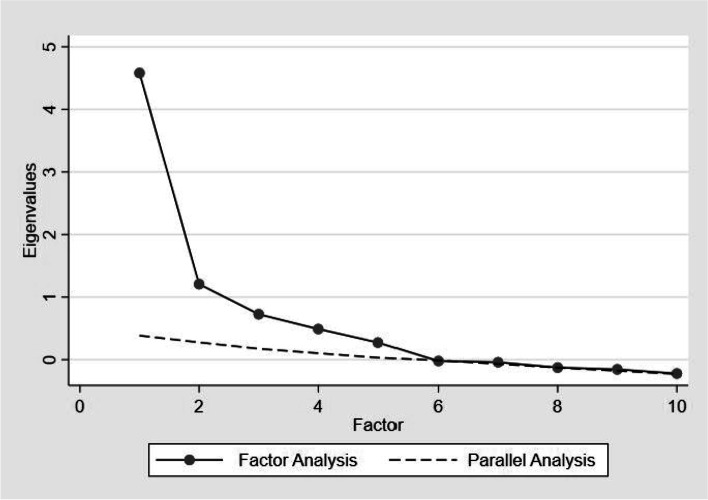
Parallel analysis depicting the number of TPB factors associated with self-management behaviour

In the primary EFA, 3 factors with Eigenvalues above 1 were found and explained 75% of the variance. Then, the numbers of factors were expanded to 4 as determined via parallel analysis (we made a decision not to maintain 5 factors are evidenced in Fig. 1 as the Eigenvalues for that particular solution fell to a value of 0.57). This model explained 84% of the variance. The KMO measure of sampling adequacy was 0.76, and the Bartlett’s test of sphericity was statistically significant (*P* < 0.01).

### Correlation between direct measures

The correlation between all direct constructs and intentions was statistically significant with a *P* value of < 0.01 (Table 2).

**Table 2:** Correlation coefficients (Pearson R) of self-management intentions and direct measures

	Population (***n*** = 266) Correlation coefficient (Pearson R)			
	Direct measures			
	Intentions	ATT	SN	PCT
**Direct measures**				
Direct attitude (ATT)		0.40^a^	–	
Direct subjective norm (SN)		0.53^a^	0.44^a^	–
Direct perceived behavioural control (PBC)		0.27^a^	0.22^a^	0.33^a^ -

^a^Correlation is significant at the 0.01 level (2-tailed)

### Direct measurements of predictive strength on intention associated with self-management behaviour

The regression analysis results indicate that the model explained 33.1% of the variance (Table 3). The model was a significant predictor of intentions associated with self-management behaviour [F (13, 266) =6.69, *P* < 0.01]. Subjective norm was the strongest predictor of intention to comply with self-management behaviour (β = 0.41, *P* < 0.01), followed by attitude ATT (β = 0.23, *P* < 0.01).

**Table 3:** Regression analysis of direct measures self-management intentions

Direct Measurements	Unstandardized Equation		Adjusted R^**2**^	ANOVA Regression Model		Collinearity Statistics	
	β	Sig		F	Sig	Tolerance	VIF
**Model**			0.37	6.69	< 0.01		
Attitude*	0.23	< 0.01				0.80	1.24
Subjective norm*	0.41	< 0.01				0.71	1.42
Perceived behaviour control*	0.05	0.50				0.75	1.33

*Adjusted for age, gender, HbA1c, BMI, income, qualifications, employment, ethnicity, total duration of living with DM (in years)

### Fitness of the model

**Fig. 2:**
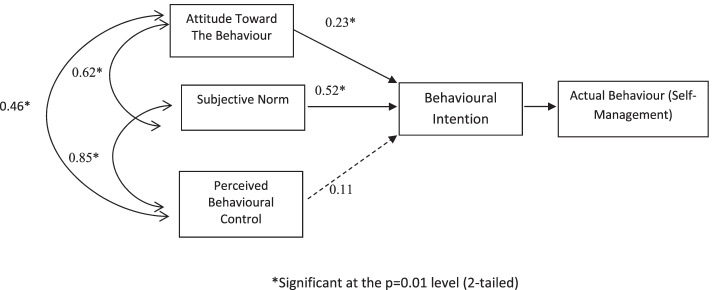
Path diagram (structural equation modelling) of TPB model of intentions associated with self-management behaviour (*regression weight is significant at the *p* < 0.01 level)

By performing confirmatory factor analysis (CFA) and SEM, the TPB model for direct measures (Fig. 2) fulfilled the indices required for a goodness of fit [root mean square error of approximation (RMSEA) < 0.01; comparative fit index (CFI) = 1.00; Tucker-Lewis index (TLI) = 1.00]. The strength of interaction between all constructs are statistically significant (β = 0.23–0.85, *P* < 0.01) except for the relationship for perceived behaviour control and intentions (β = 0.23, *P* = 0.10).

## Discussion

### Main findings

The findings from our study indicate that attitude and subjective norms are key predictors in determining the intention to follow through on self-management behaviour in people with T2D. People with T2D strongly agree that important friends and family members share the belief that they should adhere to the advice given by healthcare providers. Moreover, their willingness to comply with self-care management is influenced by two dominant factors that shape their attitude, namely 1) the relevance of self-care advice and 2) the magnitude of trust they have in the benefit of the advice given by healthcare providers. Although perceived behaviour control showed favourable psychometric properties, this construct did not hold up well to further scrutiny using more detailed statistical procedures. Nevertheless, preliminary findings from this study suggest that patient empowerment (dictated by the confidence and autonomy T2D people have over their condition) could have a critical impact on the internal locus of control pertinent to self-management behaviour.

The total variance explained of the 4-factor solution of the TPB model for self-management behaviour amongst people with T2D was excellent (> 60%) [19]. The model also demonstrated goodness of fit as evidenced by the RMSEA index (< 0.06) and CFI (> 0.95, 18]. The internal reliability of intentions and direct measures were adequate except for the perceived behavioural control. Therefore, this construct should be omitted from the TPB theoretical model. Other constructs, namely, attitude towards the behaviour and subjective norm remain important factors associated with compliance with self-management behaviour in people with T2D.

The constructs documented in this article form a minor part of a larger initiative (Integrated Behavioural Model for Diabetes Mellitus [IBM-DM]) to develop a holistic approach to identify hotspots in self-management behaviour in people with T2D [14, 15]. However, this does not imply that the whole (IBM-DM model) is greater than the sum of its part (TPB framework). By design, the TPB model described in this manuscript serves as a prelude to a more targeted screening process (the Assessment of Self-Management in Diabetes Mellitus Questionnaire [ASQ-DM]) that is currently in development [15].

### Comparison with literature

There has been considerable interest in the utility of self-management interventions in managing diabetes in the past decade, supported by the accelerated pace in the number of related publications in scientific journals [2, 7, 9]. These studies prove that by promoting self-management behaviours linked to their wishes and needs, people with T2D feel more empowered to assume an active role in their disease management [2, 7, 9]. This promising person-centered approach, in turn, has the likely potential to generate stable quality measures that can be used to benchmark overall health outcomes in these individuals.

In a similar cross-sectional study in China in 2016, the TPB framework was utilized to help identify salient beliefs surrounding oral hypoglycemic intake in people with T2D [20]. Surprisingly, the primary drivers of the TPB framework (attitude and subjective norms) were not found to be a significant element in determining medication adherence [20]. In sharp contrast to what was found in our study, barriers to perceived behavioural control were identified as the strongest predictor of intention to comply with medications [20]. Nevertheless, the authors concluded that the other constructs of the TPB remain useful in that they can align the expectations of patients with the management strategies proposed by healthcare providers [21].

From a more regional perspective, studies in Malaysia have adopted a fairly fragmented and predominantly qualitative approach to the evaluation of disease management, seeking to identify barriers to self-care in people with T2D [4, 22, 23]. For example, Thon et al. hypothesized that self-efficacy and family support were imperative to diabetes self-management [22]. Data from other studies appear to show the importance of understanding the impact on inherent perception and cultural paradigms have on people with T2D [24, 25].

These papers advocate the need to be consciously aware and cognizant of how these elements are deeply intertwined with self-management behaviour. Therefore, it is imperative to first determine the degree of commitment in people with T2D via a screening process (i.e., the determination of intention). This step can be followed by active exploration and assimilation of crucial elements (wants, needs, expectations, and obstacles) into clinical consultation, potentially resulting in a ‘win-win’ situation for both healthcare providers and patients alike [24, 25].

### Strengths and limitations

The primary strength of this study is the utility of TPB constructs as prospective screening tool to stratify the severity or urgency of intervention that could eventually necessitate a definitive diagnostic evaluation using the ASQ-DM questionnaires. By following this strategy, healthcare providers could efficiently ‘triage’ people with T2D into two broad groups that require vastly different levels of engagement or involvement. This assessment in itself creates an opportunity for a pre-emptive psychological and counselling therapy or referral.

Another strength of our study is deeply rooted in our conviction that it is possible to create a road map (PRECEDE-PROCEED model) to explain self-management behaviour in people with T2D and seamlessly integrate the concepts from this initiative into a self-regulatory health behaviour change program [13]. To that effect, the groundwork described in this manuscript sets the stage for a large-scale community engagement program, evidenced by a comprehensive assessment of the social, behavioural, environmental, and epidemiological paradigms of PRECEDE-PROCEED model [13, 26]. Essentially, this questionnaire (alongside the ASQ-DM inventory) can function as a powerful tool for a step-wise situational analysis of self-management practices in individuals with T2D, further increasing the contextual accuracy an arbitrary assessment at any point in time of the disease spectrum.

The major limitation to this study is that the data obtained from this project is exclusively cross-sectional in nature and thus necessitating the implementation of additional measures to verify the validity of the assumptions made in this manuscript. More importantly, the TPB constructs of this inventory will require a prospective determination of temporal stability to reinforce the enduring constancy of the beliefs demonstrated by participants in this study. On the other hand, we performed numerous validity assessments (SEM, parallel analysis, and EFA) to address this weakness in our study design to strengthen the value of the model conceived from the analysis of our data.

## Conclusions

A more unified approach has to be taken to identify barriers to diabetes self-management, both from a regional and global perspective. Existing psychometric questionnaires lack the statistical prowess and real-world applicability suited to the assessment of contemporary cultural sensitivity, lifestyle habits, and social narrative witnessed in people with T2D. Our research attempts to circumvent the weaknesses in research methodology by advocating a more adaptable approach to the evaluation of self-management behaviour, steep in a participatory partnership that is supported by a community engagement strategy.

In summary, this article highlights a sequential process of risk stratification via the TPB framework discussed herein (screening for intentions to comply with self-management behaviour with a specific focus on attitude and subjective norm) which could potentially enable a more detailed assessment (examination of targeted hotspots based on self-management profile) that remains a work in progress. Prospectively, these tools facilitate effective profiling and categorization of T2D people into actionable groups of high to low-risk individuals deserving a distinctive range of interventions in their own right.

## Supplementary Information

**Additional file 1.**

**Additional file 2.**

## Data Availability

The datasets generated and/or analysed during the current study are not publicly available as this information is linked closely to the unpublished research data from the IBM-DM project which is currently underway (2019–2024). However, specific/partial data will be made accessible on a reasonable request to the authors.
