# Supplementary material for: Assessing predictors of self-management intentions in people with type 2 diabetes
Source: BMC Health Serv Res. 2022 Mar 21;22:370. doi: 10.1186/s12913-022-07731-x (PMC8935112; doi:10.1186/s12913-022-07731-x)
Supplement: Supplementary file 1 — Additional file 1. [file 12913_2022_7731_MOESM1_ESM.docx]

***Questionnaire Pilot, Development And Elicitation Phase***

‘The construction of the questionnaires through a pilot study at a selected primary care clinic

80 individuals with Type 2 Diabetes (T2D) were required to answer the pilot questionnaire (direct measures), inclusive of open-ended questions (via qualitative inquiry)

Participant feedback was solicited in terms of their response to various themes which formed the basis of the development of the questionnaire

The development of an official questionnaire for formal research

***Formal Study With Final Version Of Questionnaires***

Study conducted via purposeful sampling of individuals with T2D between 2020 – 2021

Consent obtained for 417 eligible individuals with T2D who agreed to participate in the study

Participants were requested to respond to questionnaire for direct measures

All questionnaires were collected by the investigators

***Data Analysis And Interpretation Of Results***

Total of 266 participants have completed all items in the questionnaires

Internal consistency, temporal stability and correlation analysis of all constructs were conducted

Statistical analysis was conducted to evaluate the predictors of intentions to follow through on self-management parctices

**Figure 1: Flow chart of the study design and process of data collection**
