# Supplementary material for: Assessing predictors of self-management intentions in people with type 2 diabetes
Source: BMC Health Serv Res. 2022 Mar 21;22:370. doi: 10.1186/s12913-022-07731-x (PMC8935112; doi:10.1186/s12913-022-07731-x)
Supplement: Supplementary file 2 — Additional file 2. [file 12913_2022_7731_MOESM2_ESM.docx]

# Assessing Factors Related to Self-Management of Type 2 Diabetes Mellitus

This survey is part of a larger project aimed at better understanding the beliefs and attitudes that contribute to the behaviour in managing Type 2 Diabetes amongst patients. By gathering

information from many patients, we hope to learn what factors influence the intention related to the following recommended management practices in these patients.

This booklet contains a series of brief questions that take about 30 minutes to complete.

Please answer EVERY question in the booklet. Instructions on how to respond to the different questionnaires in the booklet are provided at the top of each page. Please note that there are no right or wrong answers, just what YOU think and how YOU perceive your work situation.

All the information that you provide in this session will be held in confidentiality. Your

responses will be kept by the researchers, and we will aggregate responses from all interviews so that no one individual will be identifiable.

**Background Information**

For each question below, either write in your answer in the space given.

| Age |  | | |
| --- | --- | --- | --- |
| Gender | Male Female | | |
| Race |  | Malay | |
|  |  | Indian | |
|  |  | Chinese | |
|  |  | Others | |
| Marital Status | Single Married  Divorced Widower | | |
| Work Status | Unemployed Employed (Full time)  Self Employed Employed (Part time) | | |
| Income | Individual : RM ____________  Household : RM ____________ | | |
| Education Level |  | | Certificate |
|  |  | | Diploma |
|  |  | | Bachelor’s Degree |
|  |  | | Masters |
|  |  | | PhD |
|  |  | | Others |
| Employment |  | | Unemployed |
|  |  | | Retired |
|  |  | | Labourer |
|  |  | | Technician |
|  |  | | Farming/Marine |
|  |  | | Service/Sales |
|  |  | | Business |
|  |  | | Teacher |
|  |  | | Clerk |
|  |  | | Army/Fireman/Police |
|  |  | | Manager |
|  |  | | Professional |
|  |  | | Others |
| Number of years with diabetes |  | | |
| Any other health problems |  | | |
| HbA1c levels | 1^st^ 2^nd^ 3^rd^ | | |
| BMI |  | | |

**TPB CONTRUCTS**

Directions: use the scale below to indicate how much you agree or disagree with each statement by circling the number that best corresponds to your answer in the space next to the question number. Remember there are no right or wrong answers, only what is TRUE of you.

**Generalised Intention**

| **Question Format** | **Response Format** |
| --- | --- |
| **Item 1** :  I am going to follow the instructions given during my appointment | *Strongly Disagree* **1 2 3 4 5 6 7** *Strongly Agree* |
| **Item 2** :  I want to follow the instructions given during my appointment | *Strongly Disagree* **1 2 3 4 5 6 7** *Strongly Agree* |

**Attitude**

You attend the clinic for a routine consultation. The target behaviour is following the advice or instructions given by the healthcare provider:

| **Question Format** | **Response Format** |
| --- | --- |
| **Item 3 & 4 :**  Following the instructions or advice of the doctor is | *good* ***1 2 3 4 5 6 7*** *bad*  *pleasant (for me)* ***1 2 3 4 5 6 7*** *unpleasant (for me)* |
| **Item 6 & 7 :**  Trusting the instructions or advice of the doctor during an appointment is | good **1 2 3 4 5 6 7** bad  unpleasant (for me) **1 2 3 4 5 6 7** pleasant (for me) |

**Subjective Norm**

You attend the clinic for a routine consultation. The target behaviour is following the advice or instructions given by the healthcare provider:

| **Question Format** | **Response Format** |
| --- | --- |
| **Item 9:**  Most people who are important to me think that I should follow the advice of the doctor given during my appointment at the clinic | *I should not* ***1 2 3 4 5 6 7*** *I should* |
| **Item 10:**  Everyone thinks I should follow the advice given by my healthcare provider (e.g. doctors, nurses or pharmacist) during my appointment at the clinic | *Strongly Disagree* **1 2 3 4 5 6 7** *Strongly Agree* |

**Perceived Control**

You attend the clinic for a routine consultation. The target behaviour is following the advice or instructions given by the healthcare provider:

| **Question Format** | **Response Format** |
| --- | --- |
| **Item 16:**  I am confident that I can control my disease | *Strongly Disagree* **1 2 3 4 5 6 7** *Strongly Agree* |
| **Item 17:**  It is up to me whether I can control my diabetes or not | *Strongly disagree* ***1 2 3 4 5 6 7*** *Strongly Agree* |
